# Supplementary material for: Construction of Attenuated Strains for Red-Spotted Grouper Nervous Necrosis Virus (RGNNV) via Reverse Genetic System
Source: Viruses. 2022 Aug 6;14(8):1737. doi: 10.3390/v14081737 (PMC9415089; doi:10.3390/v14081737)
Supplement: Supplementary file 1 [file viruses-14-01737-s001.zip › viruses-1783808-supplementary.pdf]

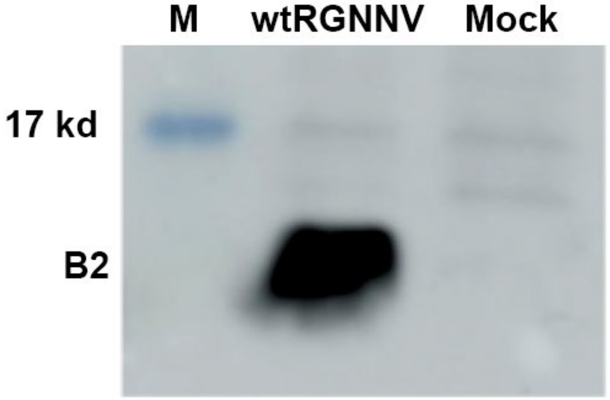

Figure S1. Immunoreactivity of B2 antibody. Positive B2 band was detected by anti-B2 polyclonal antibody in wtRGNNV infected SSN-1 cells but not in non infected SSN-1 cells (Mock).

A. Sequence of RGNNV RNA1

taacatcaccttcttgctctgttgagtaatcacttacgcaaggttaccgttcagcttagacaacgacaagtctacgccatgcgtcgctttgagtttgcactcgcacgcatgtctg
gagcagcattttgtgtgtacacaggctaccgcctgttgacctcaaaatggctcgcggaccgtgttgaggattatcgccaacgcatcatcgctgagaagaaacaaattctc
cgtgatgcggccatgatccgtactcagatccagcgggaaatggagctggtgcgcatctcggtgcgcaaaggccattcccaccaggaagctgctactgagcgtaacag
cgccactgagaccatgctcggtgtggtggagaaatgtggctacgagccatatgtcatttctccatcaccccgtgaggttggataccacgggtcacgtcagttctatagtctg
gcagattttcgccaagactaccgtcgtgatgacatcaccgaccgtcacatcattgtgatgactgatgttgactactacgtggacatgcatgagttgattggtttgggtgttccg
atattgttgtacaccttccagccaagtactgtgtccggagaggttaaggatggttactttaccatcactgacgactccgttcactaccgtgttgctggcgggaaggatgtgcg
ccaccgcatctggaactacaaccaggataccatgtatgtgtgctcccgacctcgtggtttctgggcgaatctgatgcagattctgcgtgacatcactggtgtcaccgcgatc
tgtagctttctttacaccaagctcggtattgcgccctttggcgaccctgttaccatgttcaccgttgatcaattcaagatgggtgagcatcgtaacatcgtgtcaattgtgcccttt
gcaacttgccgttcaaacctgctcaagatcagtgagtatggtgctgagttggagtatatgcgctaccaacagcgcaacaacattgccaacttcaacgcagtcacttacatc
tctgagaatgggccactcatcagtctgggtttggaaggcaattttgcaagcgtccaacttcctctgcaggatttcgagaacatccgcactgcatacgaactgtccaaaact
aacaacttgtcagatactgttcgccggtcagggcgcccgtgcaaggaggcagctatcatccataaatgtctccaagccgagtgtgccgtcgtcagcgaggtcgtgcata
aacctggcgatctcgctcgtcattaccaagcagttggtagtgcctacgacactgatccagccgagcagggcaagtgttacgcccgtgagtatgctcctggaccgttgact
caaactgctgtatttccaagtgagtcacgttccaacgagcttgccacaatcgacggtcgtattgctggtccgcaagccaaggcaaagagccgcgagcacataacacct
aagatgcgcaaagtggctagggacttcgtgcaccatctggtgccgattgccggcactggccgtccctacccccctcacgtatgtcgaggagcagcagaccaagccgtta
cagcgggctcggaatgatgctaaccgatatcacgatgagttcactatgatggtcaaagcgttccaaaagaaagaagcatacaacgccccaaattatcccaggaacatt
tcaaccgttccgcatacccaaaacgtcaagttatccagctacacctacgctttcaaagccagtgttctccagcatgttccgtggtacatgccaacgcacacaccagctga
aatcgctgacgcagtgcaaaacttggctgcaagttccactgagctggttgaaaccgactacagcaagttcgatggcacattcttgcgctttatgcgtgagtgcgtcgaattt
gctatctataagcgctgggttcacctggaccacttgccagagttaacaactttattggctaatgagatccaagcacctgctgttacacgactgggcatcaagtatgaccctg
attgcagtcgcctcagtggttctgctctcacaaccgacggaaacagcattgctaatgctttcgtctcataccttgctggtcgcatggctggcatggatgatgatgaagcttggt
cttggatcggcattgtgtacggtgatgatgggctccgatctggtaatgtttcaaacgagctcctcaccaacactgcttcttccctcggctttgacttgaagatagtgaatcgcg
cgccacgcggctctccagtgacatttctgtctcgagtatacctcgatccttggtcctcaccggcttccgtgcagtcgccattaagaacattgttgaaattgcacaccacctgtg
atacccagtcagagattgacgacattggctgggctaagacacaggcatatttggtcactgatagcaagacaccttttattggtcattggtgccgggcttatcagagaaattg
cactgcacgtgtggtccagtatgcagactacgctgacattccattctgggtgaagaacgacgaccacgttggcaactcgtggccgcagtctgaatccgatgactggaat
gacattgtagccaacgagcttggcgtcaccaccgctgagctgttgaagcatcttgcacttctggatgcttatgctggtcccatcagtggcctcccacgtctgacaacatcaa
tcgatttggaaccaaagatgtctgtcgcattagatggggagatccaagccggtcctagtcaaaacaaaactagcaaggatggaacaaatccaacaagcgatcgatca
gcacctcgtcgagctcgagcagctcttccaggtgatgatggacacgcgcgtcgctctcggcggagtgaccgcgatccaggtaaacgagatgcgcacgttcgtgataag
cgcccacgccgcagctcgccgcctacacgtcctgtcacgccggttcccaccccttccagcggtgatcgaggaaccgatggagacggactaggccgagctgctgtgcg
tcagcgtcagcgacgtcgcactcaagtgtagacaggtcacttgcctgctcccaccccttggaccgttttggtcccttaatcagctttatgctgtcctacgcttcggc

B. Sequence of RGNNV RNA2

taatccatcaccgctttgcaatcacaatggtacgcaaaggtgagaagaaattggcaaaacccgcgaccactaaggccgcgaatccgcaaccccgccgacgtgctaa
caatcgtcggcgtagtaatcgcactgacgcacctgtgtctaaggcctcgactgtaactggatttggacgtgggaccaatgacgtccatctctcaggtatgtcgagagtctcc
caggccgtcctcccagccgggacaggaacagacggatacgttgttgttgatgcaaccatcgtccccgacctcctgccacgactgggacacgctgctagaatcttccag
cgatacgctgttgaaacactggagtttgaaattcagccaatgtgccccgcaaacacgggcggtggttacgttgctggcttcctgcctgatccaactgacaacgatcacac
cttcgacgcgcttcaagcaactcgtggtgcagtcgttgccaaatggtgggaaagcagaacagtccgacctcagtacacccgcacgctcctctggacctcgtcgggaaa
ggagcagcgtctcacgtcacctggtcggctgatactcctgtgtgtcggcaacaacactgatgtggtcaacgtgtcagtgctgtgtcgctggagtgttcgactgagcgttcca
tctcttgagacacctgaagagaccaccgctcccatcatgacacaaggttccctgtacaacgattccctttccacaaatgacttcaagtccatcctcctaggatccacacca
ctggacattgcccctgatggagcagtcttccagctggaccgtccgctgtccattgactacagccttggaactggagatgttgaccgtgctgtttactggcacctcaagaagtt
tgctggaaatgctggcacacctgcaggctggtttcgctggggcatctgggacaacttcaacaagacgttcacagatggcgttgcctactactctgatgagcagccccgtc
aaatcctgctgcctgttggcactgtctgcaccagggttgactcggaaaactaaccgggtcatccggttccctagtgcgtatcgttgatgaccaattcgaacaattgattaaa
gcactaacaaatataaataaagaaatacaaacaaacaaaactgaaattggaaagaatagaagcgaaattgaaccactcgctagcaaattaaatgacaaagcaccc
aaggagggtgcgattgctattgttggtacccttgacggcgtaccggctacgcttgaaggcctatacacggctggaagcgcgccgcgtgcttaactgggtgccagtggtac
cagtcgtatccaacgccgaggaagtccctctttgggctgttgggttaccgttagctccgcgcagtgagcaccaccgccatgtggttaaatggccgctgatcgcttctcaact
gtggtt

Figure S2. Sequence of RGNNV RNA1 and RNA2.

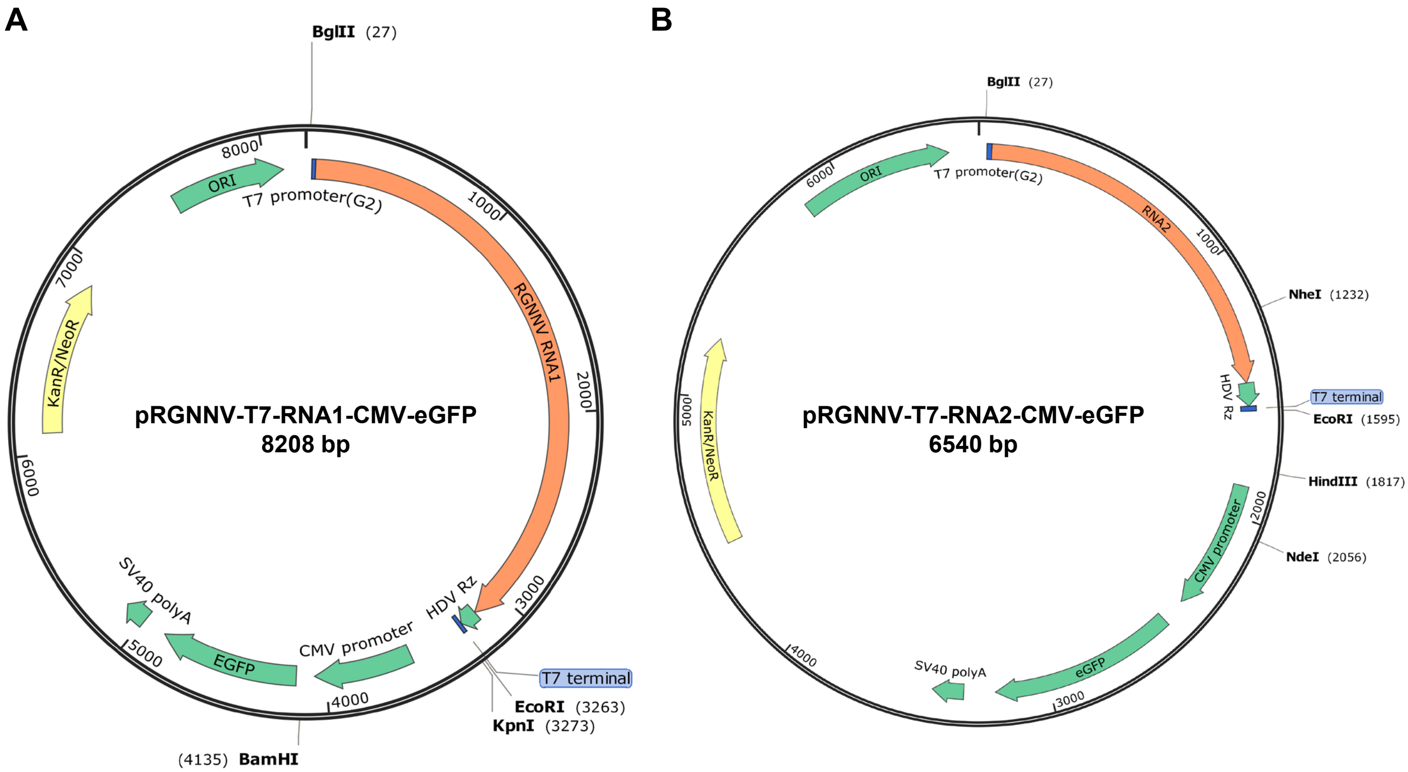

Figure S3. Plasmid maps of pRGNNV-T7-RNA1-CMV-eGFP and pRGNNV-T7-RNA2-CMV-eGFP.
